# An Investigation on the Results of Cytopathologic Tests of Pancreatobiliary System Performed in the Pathology Department in Iran

**DOI:** 10.30699/IJP.2021.131467.2462

**Published:** 2021-05-05

**Authors:** Afshin Moradi, Amir Sadeghi, Hamid Asadzadeh Aghdaei, Tahmineh Mollasharifi, Mahsa Ahadi, Elena Jamali, Afsoon Taghavi, Nasim Foroozandeh Shahraki, Arsham Moradi

**Affiliations:** 1 *Department of Pathology, School of Medicine, Shahid Beheshti University of Medical Sciences, Tehran, Iran*; 2 *Gastroenterology and Liver Diseases Research Center, Research Institute for Gastroenterology and Liver Diseases, Shahid Beheshti University of Medical Sciences, Tehran, Iran*; 3 *University of Toronto, Department of Biology, Toronto, Canada*

**Keywords:** Cytopathology, Pancreatobiliary system, ROSE, Sensitivity, Specificity

## Abstract

**Background & Objective:**

Pancreatobiliary system disorders commonly include inflammatory diseases and tumors. Diagnosis of pancreatic cancer is challenging and is mostly achieved when the disease has extensively progressed, and metastasis has occurred. Therefore, this study was performed to evaluate cytopathology in the diagnosis of Pancreatobiliary malignancies, which can improve diagnostic adequacy and accuracy.

**Methods:**

A total of 116 cytopathologic results of the Pancreatobiliary system, performed in the Pathology Department of Taleghani Hospital, Tehran, Iran during 2017-2018 were selected and examined in this observational study. The frequency of different results was determined and compared with other variables.

**Results:**

The most common location of the lesions was the pancreas (47%). The lesions were categorized as malignant, benign, negative, suspicious for malignancy (SFM), and atypical in 28%, 10%, 24%, 14%, and 9% of the cases, respectively. In other cases, lesions were considered non-diagnostic. Rapid on-site evaluation (ROSE) was conducted in 25% of patients. Compatibility of the initial and final diagnoses was 100%, 50%, and 60% in cases with “malignant”, “benign”, and “negative” diagnoses, respectively. The sensitivity, specificity, as well as positive and negative predictive values of cytopathology in the diagnosis of Pancreatobiliary lesions were 75.8%, 92.3%, 95.9%, and 61.5%, respectively.

**Conclusion:**

Our findings indicated that half of the lesions of the Pancreatobiliary system were positive, SFM, and atypical. Fine-needle aspiration (FNA) and endoscopic ultrasound-guided FNA (EUS-FNA) were effective modalities in diagnosing Pancreatobiliary malignancies. The most important point in our experience is the increase in diagnostic sensitivity in the presence of ROSE. Therefore, the simultaneous use of ROSE and EUS-FNA can reduce the need for re-sampling.

## Introduction

Pancreatobiliary system disorders often entail inflammatory diseases and tumors, which involve the pancreas, biliary tract, gallbladder, and ampullary region. Pancreatic cancer has increased in recent years with a rise in mortality by 7% amongst all deaths due to cancer in the United States and Europe making it one of the deadliest malignancies (1, 2). Several factors contribute to elevating the risk of pancreatic cancer, including smoking, overweight, obesity, exposure to certain chemicals in the workplace (e.g., benzene and petrochemicals), age, gender, race, family history, inherited genetic syndromes, diabetes, chronic pancreatitis, liver cirrhosis, gastric problems, diets, physical activities, coffee, and alcohol (3, 4).

Major reasons for delay in diagnosis of this type of cancer include non-specific symptoms associated with the disease and the proximity of major blood vessels that can be easily attacked by the tumor (5). This means that 80%-85% of tumors cannot be treated at the time of diagnosis (6). Currently, surgical removal is the only potential treatment for pancreatic cancer, while the rate of recurrence is still high, and the survival rate of patients is very low. Population-based screening for this tumor is not recommended due to the low incidence of about 1% for pancreatic cancer over the lifetime (7, 8). The International Pancreatic Cancer Screening Association suggests that people with a true definition of familial pancreatic cancer would be a potential target for screening (9). Generally, the lesions of the biliary duct and pancreas are not always readily available. Consequently, cytologic techniques are the first diagnostic method used in these cases. The use of rapid on-site evaluation (ROSE) in the cases of the pancreas has been associated with improved adequacy and diagnostic function. The ROSE procedure is performed by an appraiser (a cytopathologist or cytotechnologist) to conduct an immediate examination in terms of sampling adequacy. As a result, the accuracy of a definitive diagnosis is augmented and the need for re-sampling is diminished (10). Nowadays, brush cytology using endoscopic retrograde cholangiopancreatography (ERCP) has become the preferred method for Pancreatobiliary lesions. This technique has few side effects and allows sampling from diverse parts. The incidence of unsatisfactory samples obtained by this method is as low as about 5%. In addition, the diagnostic value of this technique is very high with very low false-positive results. The most important limitation of this method is the average reported sensitivity of 35%-48% (11). Ultrasound-guided fine-needle aspiration (EUS-FNA) is a fast, uncomplicated, accurate, and cost-effective method used to examine pancreatic tumors. Moreover, it is useful for differentiating suspected lesions from malignancies and inflammatory contributory cases. The risk of the complications of malignancy is lower than the percutaneous method (12). In 20% of cases, this technique may lead to uncertain and suspicious results due to technical or tissue issues (13). Diagnostic accuracy has increased using a new generation of needles and ultrasound devices along with immunological and molecular diagnostic methods (14). However, the simultaneous use of EUS-FNA and ROSE has raised diagnostic efficiency, while reducing time and costs (15). Benefits of ROSE encompass adequate sampling for molecular or immunohistochemistry rapid detection of malignant specimens, the possibility of multifocal tumor staging, and determination of lymph nodes involvement or distant metastasis (16). 

Various studies have been conducted in this field, including a meta-analysis of 34 studies to assess whether ROSE affects EUS-FNA diagnostic accuracy in solid pancreatic lesions. In the latter study, regression showed that ROSE was associated with EUS-FNA accuracy. Sensitivity has been reported to be 95% or higher in many studies and EUS-FNA accuracy is higher with ROSE accessibility (17). Another meta-analysis in 2014 reported that ROSE increases the EUS-FNA adequacy ratio in solid pancreatic lesions by 3%-5% (18). Klappman *et al.* (2003) compared the results of ultrasound-guided tissue sampling from two medical centers and found that ROSE increased the diagnostic function of EUS-FNA (19). A prospective single-center study in 2005 demonstrated high accuracy in a series of EUS-FNAs with ROSE (20). As a result, EUS centers were advised to be equipped with ROSE. 

Some studies have been performed exclusively on the pancreas or biliary ducts and some on biliary and ampullary lesions and pancreatic ducts, which have shown different results depending on the diagnostic methods used and the skills of the examiners. The frequency of benign, malignant, and suspicious cases was different. Furthermore, some investigations have considered suspicious cases with malignancies, while most have considered the benign ones and no separate statistics are available. Therefore, we evaluated the results of cytopathology tests of the Pancreatobiliary system performed in the Pathology Department of Taleghani Hospital, Tehran, Iran to identify the most prevalent Pancreatobiliary system disorders. Our findings might be beneficial for the timely diagnosis and treatment of lesions. Moreover, the most effective method is determined by examining different cytologic techniques in terms of the sensitivity, specificity, negative predictive value (NPV), and positive predictive value (PPV) of Pancreatobiliary lesions.

## Material and Methods

This cross-sectional analytical study was conducted to examine the cytopathologic tests of the Pancreatobiliary system performed in the Pathology Department of Taleghani Hospital in Tehran, Iran during 2017-2018. The study population entailed patients with Pancreatobiliary lesions who referred to the Pathology Department of Taleghani Hospital. A total of 116 patients were included in the present study. The inclusion criteria were the cytopathology reports of patients with the Pancreatobiliary lesion, who were first diagnosed with various sampling methods, including ERCP, EUS-FNA with or without ROSE reports and percutaneous FNA. The patients were followed by telephone, medical records, or pathology reports of the tissue sample. The final result was summarized as benign and malignant groups. The exclusion criteria encompassed not having the age or gender of the patient, lesion region, or sampling method in the report sheets.

We evaluated the reports of cytopathologic tests of patients. Furthermore, age, gender, lesion region, sample adequacy, and ROSE reports were determined and recorded in the designed information form. The lesion types were divided into six groups, namely non-diagnostic, benign, atypical, suspicious for malignancy (SFM), malignant, and negative for malignancy (21). Moreover, if present, the ROSE reports were compared with the final diagnoses (22).

In the current study, non-diagnostic cytologic specimen referred to cases not providing any proper diagnosis or information about the sampled lesion. Any cellular atypia precluded a non-diagnostic report.

Negative for malignancy were those cases, which had adequate samples without cellular atypia or evidence of malignancy. The cellularity of the specimen and extracellular tissue was sufficient for evaluation as a non-neoplastic lesion. 

The benign category indicated on those in which the cytologic specimens contained sufficient cellularity and were representative to be diagnosed as a benign neoplasm. In those cases, the smear contained predominantly acinar cells with few ductal cells. In addition, the evidence of pancreatitis (chronic, acute, and autoimmune) or pancreatic pseudocyst fell into this category.

Atypical and SFM cases were considered as an intermediate category that included a range of lesions ranging from benign cells showing minor degrees of enlargement, hyperchromia of the nucleus, or anisonucleosis (atypical) to cellular specimens demonstrating features necessary for the definitive diagnosis of carcinoma (SFM). Atypical cases were the cases with more pronounced structural and cellular aspects than reactive atypia. However, those features were less observable than what is called SFM. The related malignancy risk of the “atypical” category for EUS-FNA of solid masses was shown as 25%-100% (mean 58%) (30).

**Fig. 1:**
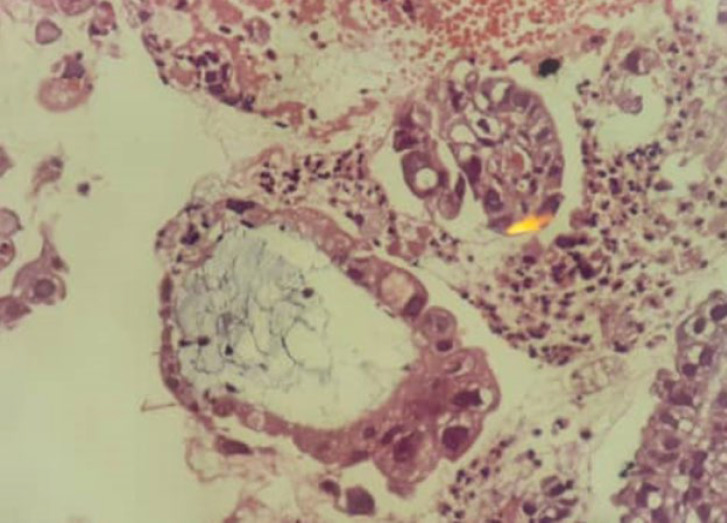
EUS-FNA, Pancreas. Cell block specimen displays a glandular structure with marked epithelial atypia, positive for malignancy

The SFM was used when some, but not all criteria of malignancy are observed, or when a very small number of cells with malignant characteristics are found. The malignancy risk associated with the diagnostic category “SFM” has been approximately reported as 82%-86% and 74% for EUS-FNA and duct brushing, respectively (30). Positive cases for malignancy included clustered cell structures, crowded plates, three-dimensional structures, micropapillae of atypical cells with high nucleus-to-cytoplasm ratio, large nucleoli with irregular nuclear membrane, and a rough chromatin pattern (Figure 1). 

The patients were followed up based on medical records, tissue pathology results, or by telephone. All data were analyzed by the Chi-square test, independent t-test, analysis of variance (ANOVA), and Bonferroni using the SPSS software version 25. The significance level was considered P-value=0.05.

## Results

The mean age of the patients was 62±13 years. Majority of the patients (60%) were male. ROSE was performed in 25% of the cases. The most common locations of the lesions were pancreas (not exactly specified) (47%) and CBD (29%) (Table 1). Lesions were positive for malignancy, benign, negative, SFM, and atypical in 28%, 10%, 24%, 14%, and 9% of the individuals, respectively. The remaining cases were classified as non-diagnostic. The sample was sufficient in 85% of cases and the sampling method was FNA in 60% of the patients. According to the Table 2, gender and lesion region were not related to the type of lesion (*P*>0.05). Furthermore, the patients with benign lesions were significantly younger than the individuals with other lesions (*P*=0.017). The findings of this study revealed that there was a statistically significant relationship between ROSE and lesion region in the studied patients (*P*=0.02) and the pancreatic lesions had a higher frequency of ROSE (Table 3). 

Table 4 represents a statistically significant relationship between sampling method and lesion type in patients evaluated in this study (*P*=0.004) and the highest rate of malignancy detection was with FNA and EUS-FNA. Table 5 displays that the sensitivity and specificity of the ROSE method were higher and lower than other techniques, respectively. Considering the positive cases and SFMs as positive cytology cases resulting from Pancreatobiliary lesions and compared to the outcomes of patients, the sensitivity and specificity of cytology in the diagnosis of Pancreatobiliary lesions were 75.8% and 92.3%, respectively. Moreover, PPV is the percentage of cases identified as positive for malignancy and SFM in cytology among the cases who were ultimately malignant in the follow-up. The NPV denotes the percentage of cases other than suspicious or positive for malignancy that was ultimately benign in the follow-up. The abovementioned factors were calculated separately for the samples for which ROSE was performed obtaining the values of 85%, 66.7%, 94.4%, and 40% for sensitivity, specificity, PPV, and NPV, respectively.

The results of the comparison of cytological results with the final results of patients in Pancreatobiliary lesions are demonstrated in Table 6. The findings showed that in malignant cases, the initial and final diagnoses were 100% matched. However, the cases reported as benign and negative for malignancy were 50% and 60% matched the final results as benign, which could indicate the acceptable PPV for cytology versus its NPV. In the re-examination, 70% of the case in the non-diagnostic category were malignant, which shows the importance of follow-up.

Further analysis was performed on diverse variables considered as the source of confounder factors shown in Tables 7 and 8. The gender variable had no significant contribution to other variables (Table 7). It was previously found that the patients with benign lesions were significantly younger than others. We evaluated the correlation of age with other variables and observed that patients with lesions in the pancreas body were younger than others (*P*=0.017). However, the correlation of age with other variables was not found to be statistically significant (Table 8).

**Table 1:** The distribution of different variables in the patients

Variables		Mean (Frequency)
Gender	Male	70 (60.3%)
	Female	46 (39.7%)
ROSE	No	87 (75.0%)
	Yes	29 (25.0%)
Lesion location	Pancreas	55 (47.4%)
	Pancreatic head	10 (8.6%)
	Pancreatic tail	2 (1.7%)
	Common bile duct	34 (29.3%)
	Liver hilum	3 (2.6%)
	Site not specified	2 (1.7%)
	Pancreatic Body	3 (2.6%)
	Gallbladder	3 (2.6%)
	Ampulla of Vater	4 (3.4%)
Lesion type	Malignant	33 (28.4%)
	Benign	12 (10.3%)
	Suspicious	16 (13.8%)
	Negative	28 (24.1%)
	Non-Diagnostic	17 (14.7%)
	Atypical	10 (8.6%)
Specimen type	Sufficient	99 (85.3%)
	Insufficient	17 (14.7%)
Sampling method	FNA	69 (59.5%)
	Brush	38 (32.8%)
	EUS-FNA	9 (7.8%)

**Table 2:** The relationship between the type of lesion, age, gender, and the location of the lesions in the subjects

Variables		Malignant	Benign	Suspicious	Negative	Non Diagnostic	Atypical	P- value
		Mean (frequency)						
Age (Mean ± SD)		61.94 ± 13.57	51.58 ± 8.68	62.81 ± 11.64	61.43 ± 11.7	64.35 ± 15.39	71.3 ± 10.82	0.017
Gender	Male	18 (54.5%)	7 (58.3%)	11 (68.8%)	20 (71.4%)	7 (41.2%)	7 (70.0%)	0.370
	Female	15 (45.5%)	5 (41.7%)	5 (31.3%)	8 (28.6%)	10 (58.8%)	3 (30.0%)	
Lesion Location	Pancreas(not specified)	21 (63.6%)	9 (75.0%)	9 (56.3%)	12 (42.9%)	3 (17.6%)	1 (10.0%)	0.072
	Pancreatic head	4 (12.1%)	2 (16.7%)	0 (0.0%)	0 (0.0%)	2 (11.8%)	2 (20.0%)	
	Pancreatic tail	2 (6.1%)	0 (0.0%)	0 (0.0%)	0 (0.0%)	0 (0.0%)	0 (0.0%)	
	Common bile duct	4 (12.1%)	1 (8.3%)	5 (31.3%)	10 (35.7%)	9 (52.9%)	5 (50.0%)	
	Liver hilum	0 (0.0%)	0 (0.0%)	0 (0.0%)	2 (7.1%)	0 (0.0%)	1 (10.0%)	
	Site not specified	0 (0.0%)	0 (0.0%)	1 (6.3%)	1 (3.6%)	0 (0.0%)	0 (0.0%)	
	Pancreatic Body	1 (3.0%)	0 (0.0%)	0 (0.0%)	1 (3.6%)	1 (5.9%)	0 (0.0%)	
	Gallbladder	0 (0.0%)	0 (0.0%)	1 (6.3%)	1 (3.6%)	0 (0.0%)	1 (10.0%)	
	Ampulla of vater	1 (3.0%)	0 (0.0%)	0 (0.0%)	1 (3.6%)	2 (11.8%)	0 (0.0%)	

**Table 3:** Relationship between ROSE and lesion location in the patients

Lesion Location	ROSE		P-value
	No	Yes	
Pancreas )not specified)	35 (40.2%)	20 (69.0%)	0.02
Pancreatic head	6 (6.9%)	4 (13.8%)	
Pancreatic tail	2 (2.3%)	0 (0.0%)	
Common bile duct	32 (36.8%)	2 (6.9%)	
Liver hilum	3 (3.4%)	0 (0.0%)	
Site not specified	2 (2.3%)	0 (0.0%)	
Pancreatic Body	1 (1.1%)	2 (6.9%)	
Gallbladder	2 (2.3%)	1 (3.4%)	
Ampulla of Vater	4 (4.6%)	0 (0.0%)	

**Table 4:** The relationship between sampling method and type of lesion in patients

Lesion Type	Sampling Type			P-value
	FNA	Brush	EUS-FNA	
Malignant	26 (37.7%)	4 (10.5%)	3 (33.3%)	0.004
Benign	8 (11.6%)	1 (2.6%)	3 (33.3%)	
Suspicious	11 (15.9%)	5 (13.2%)	0 (0.0%)	
Negative	14 (20.3%)	12 (31.6%)	2 (22.2%)	
Non-Diagnostic	5 (7.2%)	11 (28.9%)	1 (11.1%)	
Atypical	5 (7.2%)	5 (13.2%)	0 (0.0%)	

**Table 5:** Cytological diagnostic specificity and sensitivity for pancreatobiliary lesions and positive and negative cytological predictive value

	Sensitivity (%)	Specificity (%)	PPV	NPV
Cytology (all data)	75.8	92.3	95.9	61.5
Cytology (ROSE +)	85	66.7	94.4	40

**Table 6:** Comparison of cytological results with the final results of the patients in pancreatobiliary lesions

			Total	Final result		P-value
				Malignant	Benign	
All Data	Cytology	Malignant	33	33 (100.0%)	0 (0.0%)	<0.001
		Benign	12	6 (50.0%)	6 (50.0%)	
		Suspicious	16	13 (81.3%)	3 (18.8%)	
		Negative	28	11 (39.3%)	17 (60.7%)	
		Non-Diagnostic	17	12 (70.6%)	5 (29.4%)	
		atypical	10	4 (40.0%)	6 (60.0%)	
ROSE +	Cytology	Malignant	15	15 (100.0%)	0 (0.0%)	0.002
		Benign	2	1 (50.0%)	1 (50.0%)	
		Suspicious	4	2 (50.0%)	2 (50.0%)	
		Negative	5	3 (60.0%)	2 (40.0%)	
		Non-Diagnostic	1	0 (0.0%)	1 (100.0%)	
		atypical	2	2 (100.0%)	0 (0.0%)	

**Table 7:** Subgroup analysis (considering sex variable)

		Sex				P-value
		Male		Female		
Lesion Location	Pancreas	35 (63.6%)		20 (36.4%)		0.433
	Pancreatic head	7 (70.0%)		3 (30.0%)		
	Pancreatic tail	0 (0.0%)		2 (100.0%)		
	Common bile duct	22 (64.7%)		12 (35.3%)		
	Liver hilum	1 (33.3%)		2 (66.7%)		
	Site not specified	1 (50.0%)		1 (50.0%)		
	Pancreatic Body	1 (33.3%)		2 (66.7%)		
	Gallbladder	2 (66.7%)		1 (33.3%)		
	Ampulla of Vater	1 (25.0%)		3 (75.0%)		
Cytology result	Malignant	31 (59.6%)		21 (40.4%)		0.473
	Other	30 (66.7%)		15 (33.3%)		
Specimen type	Sufficient	63 (63.6%)		36 (36.4%)		0.08
	Insufficient	7 (41.2%)		10 (58.8%)		
Sampling method	FNA	42 (60.9%)		27 (39.1%)		0.954
	Brush	23 (60.5%)		15 (39.5%)		
	EUS-FNA	5 (55.6%)		4 (44.4%)		
Final result	Malignant	48 (60.8%)		31 (39.2%)		0.894
	Benign	22 (59.5%)		15 (40.5%)		

## Discussion

Pancreatobiliary lesions are one of the most important types of GI lesions that are not always easily available for biopsy. Therefore, cytological techniques are the first diagnostic method used in these cases. Some studies have been performed only on the pancreas or biliary ducts and some on biliary and ampullary lesions. The results have been different depending on the used diagnostic methods, the skills of examiners, and the frequency of benign, malignant, or suspicious cases. Moreover, some publications have considered suspicious cases with malignancies, while most of them have considered benign ones without separate statistics.

In the present study, the most common location of the lesions were the pancreas (47%), followed by CBD (29%). The lesions were malignant, benign, negative for malignancy, SFM, and atypical in 28%, 10%, 24%, 14%, and 9% of the patients, respectively. The remaining cases were non-diagnostic. In addition, ROSE was carried out in 25% of the cases. Gender and lesion region were not related to the type of lesion. However, the patients with benign lesions were significantly younger than the individuals with other lesions (*P*=0.017). There was a statistically significant relationship between sampling method and lesion type in patients examined in this study (*P*=0.004) and the highest rate of malignancy detection was with FNA and EUS-FNA. In an intervention conducted by Gábor Elek *et al.*, a total of 205 specimens obtained by brush cytology and biopsy from 113 patients with Wirsung’s duct stenosis were examined. In the latter study, 103 cases were diagnosed during surgery, autopsy, or based on the clinical course of patients (21). Diagnostic accuracy before surgery depended on the location of the tumor, which was more common in ampullary and parapapillary tumors. The mean sensitivity, specificity, PPV, NPV, and cytology accuracy were 53%, 100%, 100%, 25%, and 59%, respectively. However, the mean sensitivity, specificity, PPV, and NPV in our study for Pancreatobiliary cytology were 75.8%, 92.3%, 95.9%, and 61.5%, respectively. The corresponding values for biopsy were 43%, 100%, 100%, 36%, and 56%, respectively. Close collaboration with the endoscopist was required in 27% of the samples where the sample size was insufficient or undetectable. Among the 26 false-negative samples, 84%, 4%, and 12% of the cases had sampling errors, technical errors, and findings interpretation issues, respectively (23). In our study, nearly one-sixth of the specimens were non-diagnostic, 70% of which were malignant in the follow up indicating the need to use better and more accurate sampling methods, such as ROSE.

**Table 8:** Subgroup analysis (considering age variable)

		Mean ± SD	P-value
Lesion Location	Pancreas	60.64 ± 12.52	0.017
	Pancreatic head	61.2 ± 11.89	
	Pancreatic tail	61 ± 0	
	Common bile duct	65.76 ± 10.51	
	Liver hilum	72.33 ± 15.14	
	Site not specified	53 ± 26.87	
	Pancreatic Body	37.33 ± 20.11	
	Gallbladder	60.67 ± 19.86	
	Ampulla of vater	68.25 ± 10.87	
Cytology result	Malignant	63.29 ± 13.21	0.086
	Other	58.91 ± 11.33	
Specimen type	Sufficient	61.63 ± 12.65	0.428
	Insufficient	64.35 ± 15.39	
Sampling method	FNA	60.51 ± 13.67	0.061
	Brush	65.47 ± 11.68	
	EUS-FNA	59.11 ± 11.92	
Final result	Malignant	63.24 ± 12.3	0.144
	Benign	59.43 ± 14.35	

In a study done by Stoos-Veic *et al.* on 143 brushing samples, 25% of cases were malignant cytologically, 63.6% were benign, and other cases were considered suspicious. Moreover, it was found that 20 of the negative results were false. By removing the atypical or suspicious cases, sensitivity and specificity were calculated as 64% and 100%, respectively. Consequently, considering them as real positives, the sensitivity increased up to 71%. The authors found that gallbladder brushing could be valuable for diagnosing Pancreatobiliary pathologies. However, it highly depends on the skill of the endoscopist and cytologist (24). It could be compared with the PPV of 95.5% in our study. In an investigation by Yamaguchi *et al.*, two pathologists examined 127 patients with pancreatic ductal adenocarcinoma (PDAC) and 74 individuals with benign pancreatic duct stricture mimicking PDAC. The final diagnosis was confirmed based on histopathology by resection or over 1 year of follow-up. Pancreatic juice cytology (PJC) was examined before and after brush cytology. In those with PDAC, the sensitivity of PJC before and after brushing was 21.3% and 40.9%, respectively. Furthermore, it was 48.8% for BC. Out of 65 patients with PDAC, in whom neither PJC before brushing nor BC indicated malignancy, 16 were diagnosed with pancreatic cancer using PJC after brushing. Brush cytology combined with PJC after brushing significantly raised the diagnostic sensitivity for PDAC to 61.4%. Therefore, BC combined with PJC after brushing was more reliable than PJC before brushing or BC for the diagnosis of pancreatic cancer. In our study, based on the available resources, grouping into six diagnostic cytology categories was used, which elevated the observed diagnostic efficiency and sensitivity of cytology for the diagnosis of Pancreatobiliary malignancies to 75.8%.

Mahmoudi *et al.* (2008) completed a cross-sectional study on 199 cytological brush samples and showed 77 patients (41%) with positive results for malignancy (25). Result-related variables included age, mass size over 1 cm, and stricture length over 1 cm. Sensitivity, specificity, PPV, and NPV were 61%, 98%, 99%, and 57%, respectively. In the current study, age had a significant relationship with cytologic findings, and in benign cases, the patients were younger.

In China, Yang *et al.* in 2019 reported that the ROSE method is accurate and helpful for detecting lesions in the Pancreatobiliary system while reducing the detection time in this group of patients. In addition, it can indicate the need for subsequent sampling if necessary (13). In our study, ROSE was performed in 25% of cases and augmented the sensitivity of cytology to 80%. 

Conti *et al.* (2019) in Italy reported that each of the FNB or FNA methods had good diagnostic efficacy when used for pancreatic tumors, and the decision to use any of these methods depends on the condition of the patient and the opinion of the physician (26). Of course, in our study, the results obtained from one of the FNA, EUS-FNA, or brush methods were used for each patient, while the highest rate of malignancy detection was achieved by FNA and EUS-FNA. Pitman *et al.* (2014) suggested EUS-FNA as the selected method of sampling for the diagnosis of pancreatic malignancy (27). The results of this study were in line with our investigation. Furthermore, Pitman *et al.* provided new classifications for standardized terminology and nomenclature that was effective in predicting biological behavior and recommendation management. In the present study, we performed the same six-group classification.

A review in Spain by Iglesias *et al.* (2014) showed that the use of ROSE elevated the diagnostic efficiency of pancreas masses. However, this increase was to the extent of 10%-30% and was more common in referral hospitals where the time of the procedure is important, and the usual diagnostic accuracy is < 90%. They concluded that the use of ROSE should be limited to such centers (28). However, in our research, the diagnostic efficiency of ROSE was appropriate making it very helpful.

In a meta-analysis and a systematic review of 1299 patients with pancreatic lesions, it was found that the application of ROSE would not affect diagnostic efficiency. In the mentioned study the sensitivity and specificity of ROSE were 91% and 100%, respectively. In the absence of ROSE, sensitivity and specificity obtained for patients were 85% and 100%, respectively (29). Furthermore, Cozo *et al.* (2015) reported 62.4% sensitivity and 97.7% specificity for cytology and in the diagnosis of Pancreatobiliary malignancies. The addition of CA-19.9 and CA-125 increased diagnostic sensitivity up to 94.1% (31). However, the sensitivity and specificity of cytopathology for the diagnosis of Pancreatobiliary malignancy were 75.8% and 92.3%, respectively. The addition of ROSE elevated diagnostic sensitivity to 80%. We observed a statistically significant relationship between ROSE and the location of the lesion in the studied patients (*P*=0.02) with the pancreatic lesions having a higher frequency of ROSE procedure. The need for ROSE remains one of the most contentious issues in the EUS-FNA. Experts recommend that ROSE can be performed in centers with a sufficient staff of cell therapists (32). 

##  Conclusion

According to the results of the current study, more than half of the lesions of the Pancreatobiliary system could be interpreted as positive, SFM, and atypical. Therefore, it is very important to study and take into consideration these lesions from a diagnostic and therapeutic perspective. This analysis showed that FNA and EUS-FNA would be effective modalities in diagnosis of Pancreatobiliary malignancies. Further multicenter studies with a larger sample size are needed to confirm the findings of the present study. In addition, investigation of the role of complementary diagnostic methods is recommended for future studies. Moreover, it is suggested to reduce the incidence of biliary and pancreatic malignancies by taking the necessary precautions. The most important point in our experience was the elevated diagnostic sensitivity in the presence of on-site cytopathology assessment. Consequently, simultaneous use of ROSE and EUS-FNA methods would prevent having inadequate samples and reduce the need for re-sampling.

## References

[1] Ettinghausen SE, Schwartzentruber DJ, Sindelar WF (1995). Evolving strategies for the treatment of adenocarcinoma of the pancreas A review. J Clin Gastroenterol.

[2] Levy MJ, Wiersema M, Chari ST (2006). Chronic pancreatitis: focal pancreatitis or cancer? Is there a role for FNA/biopsy? Autoimmune pancreatitis. Endoscopy.

[3] Thomas C, Ngheim H, Pellegrini C (1996). Pancreatic cancer diagnosis and therapy. Cancer of the Pancreas.

[4] Bray F, Ren JS, Masuyer E, Ferlay J (2013). Global estimates of cancer prevalence for 27 sites in the adult population in 2008. Int J Cancer.

[5] Kocjan G, Smith AN (1997). Bile duct brushings cytology: potential pitfalls in diagnosis. Diagn Cytopathol.

[6] Siddiqui AA, Brown LJ, Hong S-KS, Draganova-Tacheva RA, Korenblit J, Loren DE (2011). Relationship of pancreatic mass size and diagnostic yield of endoscopic ultrasound-guided fine needle aspiration. Digest Dis Sci.

[7] Logrono R, Kurtycz DF, Molina CP, Trivedi VA, Wong JY, Block KP (2000). Analysis of false-negative diagnoses on endoscopic brush cytology of biliary and pancreatic duct strictures: the experience at 2 university hospitals. Arch pathol Lab Med.

[8] Navaneethan U, Njei B, Lourdusamy V, Konjeti R, Vargo JJ, Parsi MA (2015). Comparative effectiveness of biliary brush cytology and intraductal biopsy for detection of malignant biliary strictures: a systematic review and meta-analysis. Gastrointest Endosc.

[9] Adhya AK, Kar M, Mohanty R (2018). Diagnostic utility of touch imprint cytology in the evaluation of intra abdominal tumors. Oncol J India.

[10] Bruno P, Ricci A, Esposito M, Scozzi D, Tabbì L, Sposato B (2013). Efficacy and cost effectiveness of rapid on site examination (ROSE) in management of patients with mediastinal lymphadenopathies. Eur Rev Med Pharmacol Sci.

[11] Varadarajulu S, Tamhane A, Eloubeidi MA (2005). Yield of EUS-guided FNA of pancreatic masses in the presence or the absence of chronic pancreatitis. Gastrointest Endosc.

[12] Levy MJ, Clain JE, Clayton A, Halling KC, Kipp BR, Rajan E (2007). Preliminary experience comparing routine cytology results with the composite results of digital image analysis and fluorescence in situ hybridization in patients undergoing EUS-guided FNA. Gastrointest Endosc.

[13] Yang F, Liu E, Sun S (2019). Rapid on-site evaluation (ROSE) with EUS-FNA: The ROSE looks beautiful. Endosc Ultrasound.

[14] Yamaguchi T, Shirai Y, Nakamura N, Sudo K, Nakamura K, Hironaka S (2012). Usefulness of brush cytology combined with pancreatic juice cytology in the diagnosis of pancreatic cancer: significance of pancreatic juice cytology after brushing. Pancreas.

[15] Kong F, Kong X, Zhu J, Sun T, Du Y, Wang K (2019). A prospective comparison of conventional cytology and digital image analysis for the identification of pancreatic malignancy in patients undergoing EUS-FNA. Endosc Ultrasound.

[16] Al-Haddad M, Eloubeidi MA (2010). Interventional EUS for the diagnosis and treatment of locally advanced pancreatic cancer. J Pancreas.

[17] Hebert‐Magee S, Bae S, Varadarajulu S, Ramesh J, Frost A, Eloubeidi M (2013). The presence of a cytopathologist increases the diagnostic accuracy of endoscopic ultrasound‐guided fine needle aspiration cytology for pancreatic adenocarcinoma: a meta‐analysis. Cytopathology.

[18] Khurana KK, Graber B, Wang D, Roy A (2014). Telecytopathology for on-site adequacy evaluation decreases the nondiagnostic rate in endoscopic ultrasound-guided fine-needle aspiration of pancreatic lesions. Telemed J E Health.

[19] Klapman JB, Logrono R, Dye CE, Waxman I (2003). Clinical impact of on-site cytopathology interpretation on endoscopic ultrasound-guided fine needle aspiration. Am J Gastroenterol.

[20] Tournoy KG, Praet MM, Van Maele G, Van Meerbeeck JP (2005). Esophageal endoscopic ultrasound with fine-needle aspiration with an on-site cytopathologist: high accuracy for the diagnosis of mediastinal lymphadenopathy. Chest.

[21] Elek G, Gyökeres T, Schäfer E, Burai M, Pintér F, Pap Á (2005). Early diagnosis of pancreatobiliary duct malignancies by brush cytology and biopsy. Pathol Oncol Res.

[22] Štoos-Veić T, Bilić B, Kaić G, Trutin Ostović K, Babić Ž, Kujundžić M (2010). Biliary brush cytology for the diagnosis of malignancy: a single center experience. Coll Antropol.

[23] Levy MJ, Oberg TN, Campion MB, Clayton AC, Halling KC, Henry MR (2012). Comparison of methods to detect neoplasia in patients undergoing endoscopic ultrasound-guided fine-needle aspiration. Gastroenterology.

[24] Vincent A, Herman J, Schulick R, Hruban RH, Goggins M (2011). Pancreatic cancer. The lancet.

[25] Mahmoudi N, Enns R, Amar J, AlAli J, Lam E, Telford J (2008). Biliary brush cytology: factors associated with positive yields on biliary brush cytology. World J Gastroenterol: WJG.

[26] Conti CB, Cereatti F, Grassia R (2019). Endoscopic ultrasound-guided sampling of solid pancreatic masses: the fine needle aspiration or fine needle biopsy dilemma Is the best needle yet to come?. World J Gastroenterol.

[27] Pitman MB, & Layfield LJ (2014). Guidelines for Pancreatobiliary cytology from the Papanicolaou Society of Cytopathology: a review. Cancer Cytopathol.

[28] Iglesias-Garcia J, Lariño-Noia J, Abdulkader I, Domínguez-Muñoz JE (2014). Rapid on-site evaluation of endoscopic-ultrasound-guided fine-needle aspiration diagnosis of pancreatic masses. World J Gastroenterol.

[29] Kong F, Zhu J, Kong X, Sun T, Deng X, Du Y (2016). Rapid on-site evaluation does not improve endoscopic ultrasound-guided fine needle aspiration adequacy in pancreatic masses: a meta-analysis and systematic review. PLoS One.

[30] Martha Bishop Pitman, Lester James Layfield (2015). The Papanicolaou Society of Cytopathology System for Reporting Pancreatobiliary Cytology.

[31] Kuzu UB, Ödemiş B, Turhan N, Parlak E, Dişibeyaz S, Suna N, Torun S (2015). The diagnostic value of brush cytology alone and in combination with tumor markers in Pancreatobiliary strictures. Gastroenterol Res Pract.

[32] Yang F, Liu E, & Sun S (2019). Rapid on-site evaluation (ROSE) with EUS-FNA: The ROSE looks beautiful. Endoscopic ultrasound.

